# Supplementary material for: Machine Learning Approach for Muscovy Duck (Cairina moschata) Semen Quality Assessment
Source: Animals (Basel). 2023 May 10;13(10):1596. doi: 10.3390/ani13101596 (PMC10215291; doi:10.3390/ani13101596)
Supplement: Supplementary file 1 [file animals-13-01596-s001.zip › animals-2298495-supplementary.pdf]

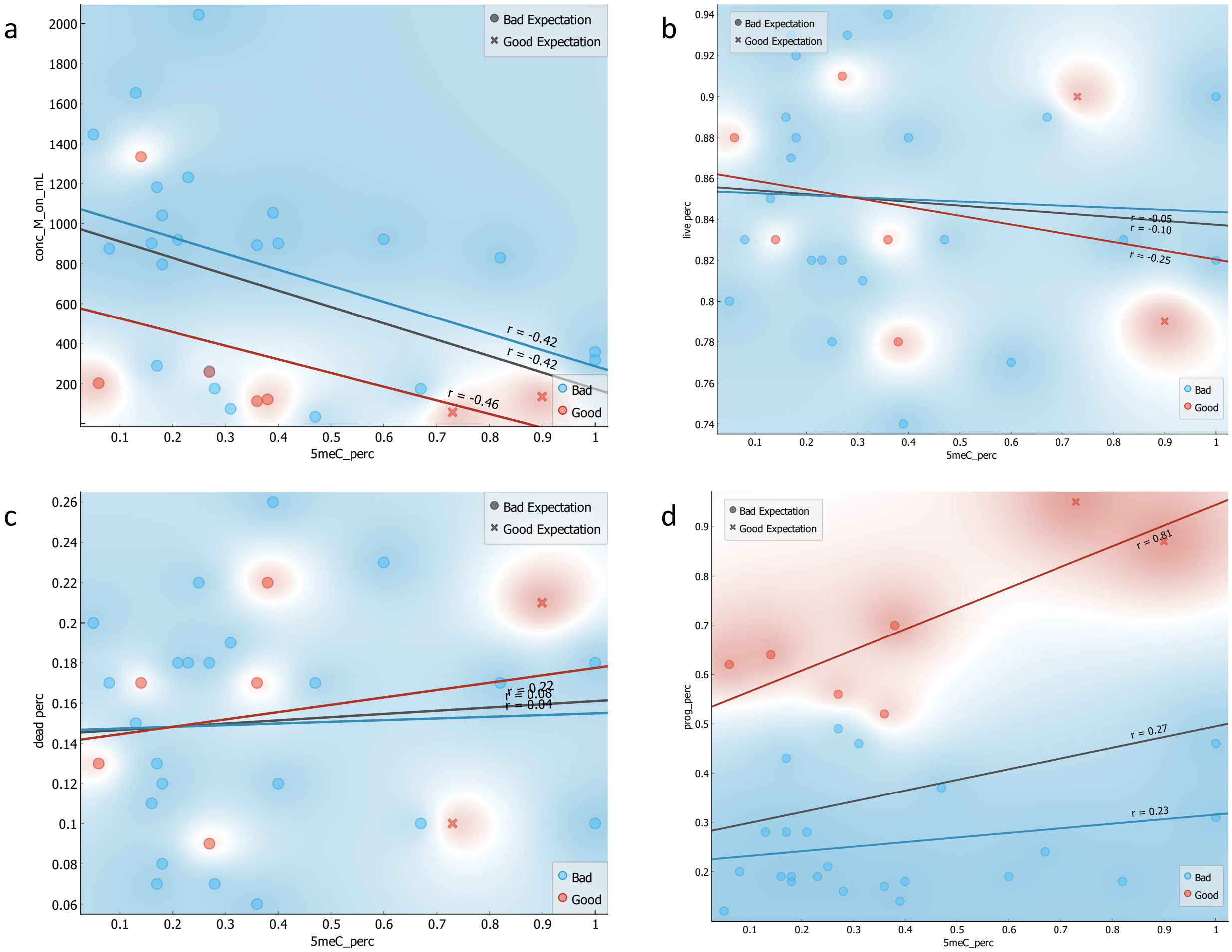

Supplementary Figure S1. *Correlation plots of 5meC (x-axis) and sperm concentration (a), percentage of live cells stained with BrightVit (b), percentage of dead cells stained with BrightVit (c), and progressive motility percentage (d).*

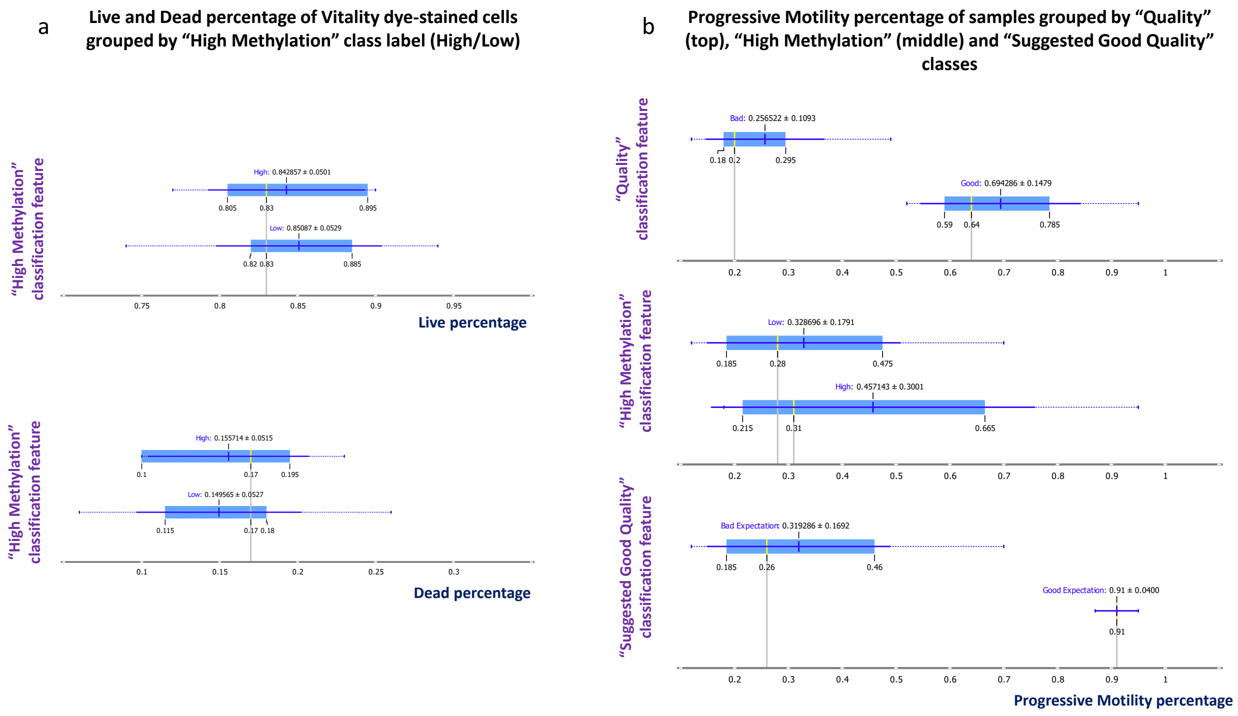

***Supplementary Figure S2**. a) Box-plots showing annotation for end values, the mean and the median, standard deviations and quantiles of Live Vitality Dye Cell staining percentage (top) and Dead Vitality Dye Cell staining percentage in class “High Methylation” (bottom). b) Box-plots showing annotation for end values, the mean and the median, standard deviations and quantiles of Progressive Motility percentages in classes “Quality” (top), “High Methylation” (middle), and “Suggested Good Quality” (bottom). The **mean** is depicted as dark blue vertical line. The thin blue line represents the **standard deviation**. The blue highlighted area represents the values between the first and the third **quartile**. Values of the first (25%) and the third (75%) quantile are presented on both sides of this area. The **median** is depicted as an yellow vertical line.*

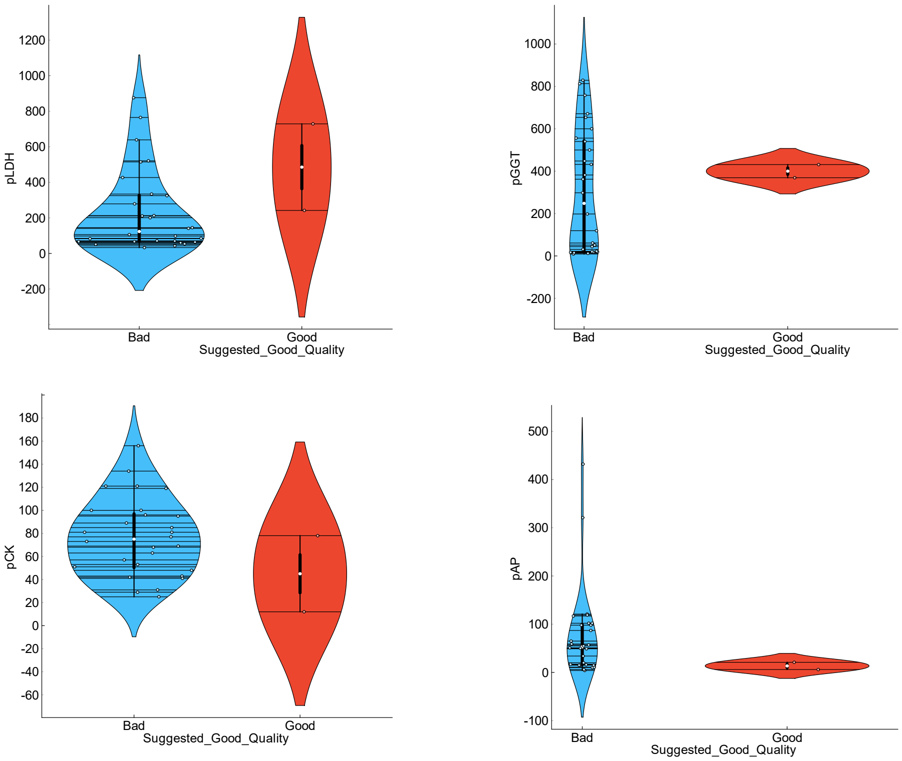

Supplementary Figure S3. *Violin plots of enzyme activities (LDH, GGT, CK, AP) in category Suggested Quality*

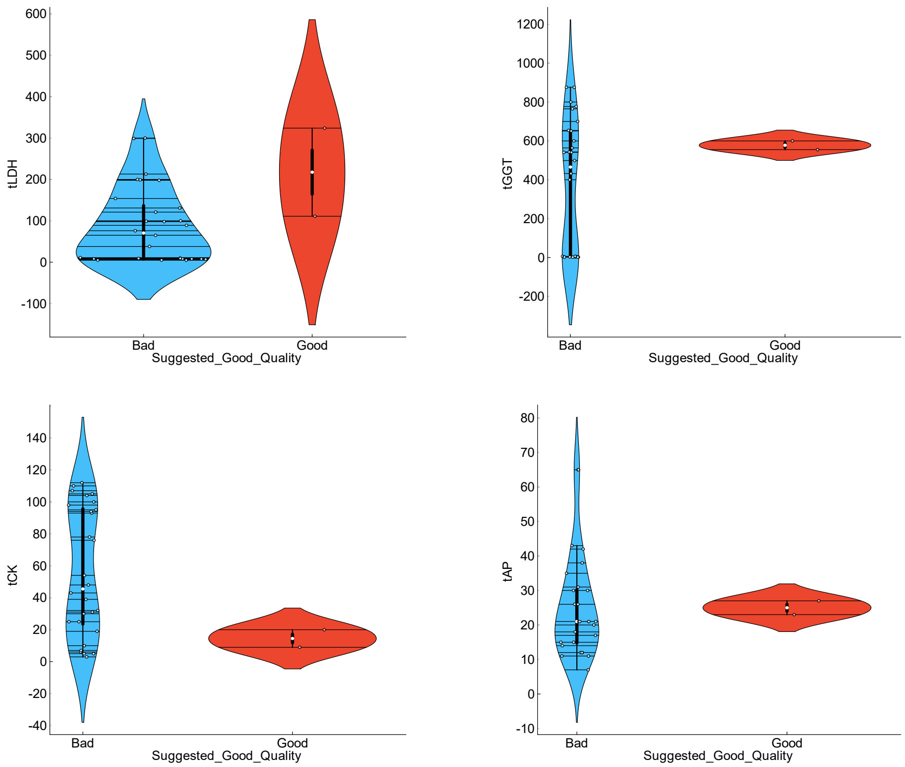

Supplementary Figure S4*. Violin plots of enzyme activities (LDH, GGT, CK, AP) in category "Suggested Good Quality"*

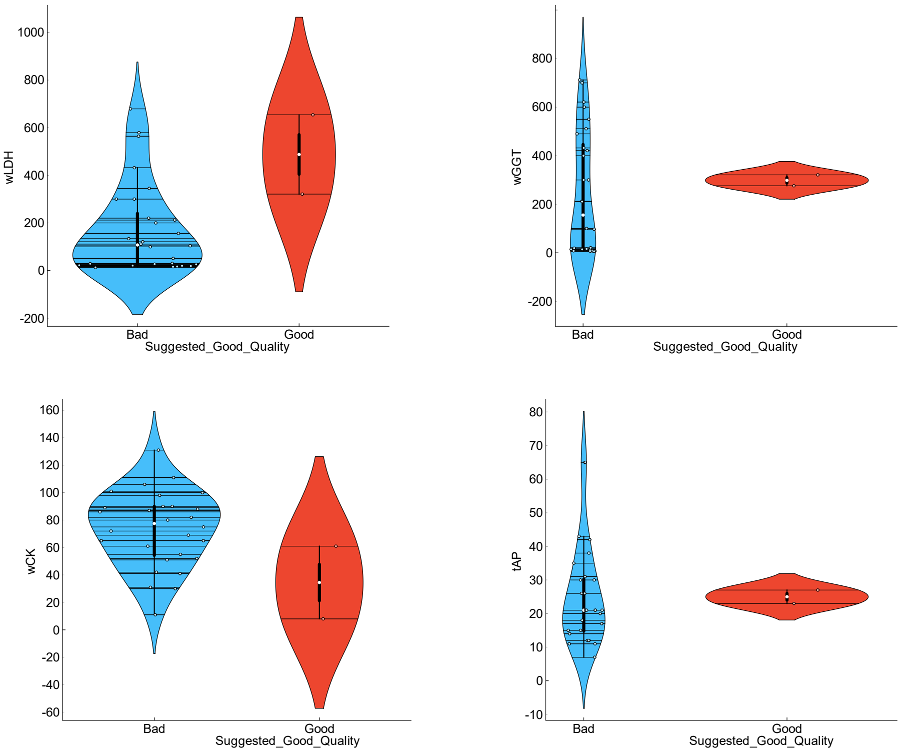

Supplementary Figure S5. *Violin plots of enzyme activities (LDH, GGT, CK, AP) in category "Suggested Good Quality"*

Table S1. ML Classifiers on Quality Parameter – Best model describing Quality

Model	AUC	CA	F1	Precision	Recall
**Neural Network**	**0.944**	**0.867**	**0.858**	**0.861**	**0.867**
**Naive Bayes**	**0.932**	**0.667**	**0.69**	**0.863**	**0.667**
**SVM**	**0.925**	**0.8**	**0.787**	**0.784**	**0.8**
Random Forest	0.845	0.833	0.829	0.826	0.833
kNN	0.82	0.8	0.8	0.8	0.8
Extreme Gradient Boosting Random Forest (xgboost)	0.733	0.9	0.897	0.897	0.9
Gradient Boosting (scikit-learn)	0.727	0.833	0.829	0.826	0.833
AdaBoost	0.72	0.8	0.8	0.8	0.8

Table S2. Top 5 Model Feature ranked based on Models predicting "Quality" classification feature (label)

	Gain ratio	Gini	ANOVA	ReliefF
VCL	0.320	0.299	61.963	0.247
ALH	0.215	0.199	45.324	0.234
tGGT	0.144	0.110	10.892	0.227
VAP	0.164	0.138	21.755	0.170
tCK	0.144	0.110	9.276	0.143

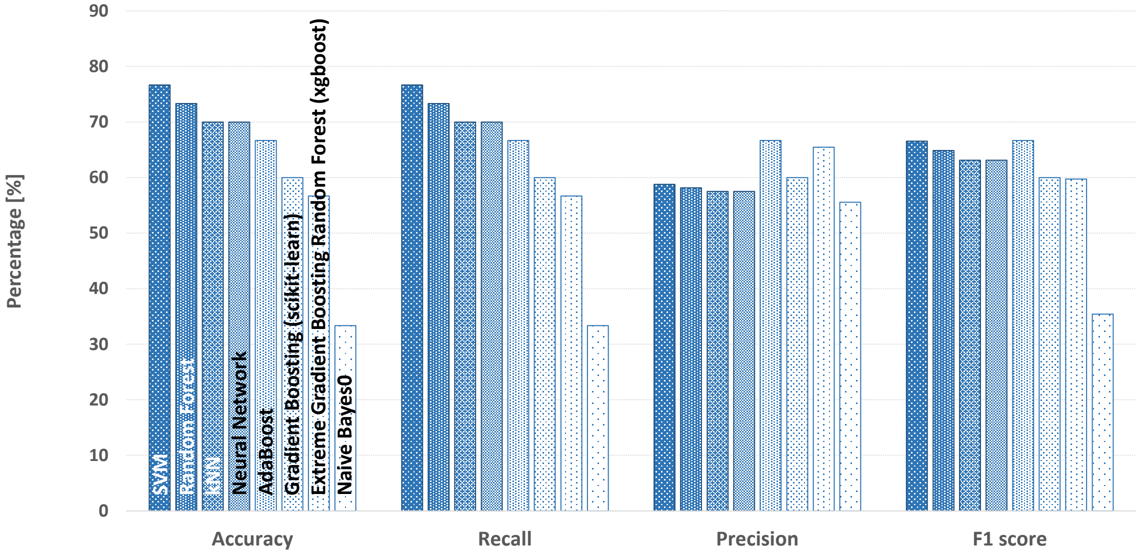

Supplementary Figure S6. *Classification power of the learning algorithms used to classify the sperm dataset based on classifying label "High Methylation". Predictions were made on dataset in 5-fold cross-validation process, where total dataset was split into 5 folds, and each was once used as a test sets, while remaining were used as validation set. Each cross-validation repetition used a different seed. Metrics presented are Datamining package "Orange" cross-validation algorithm performance parameters. a) The percentage accuracy, recall, precision, and F1-score of the different ML algorithm models are compared with each other. None of the ML algorithms does not perform well enough for proper classification of the data.*

Table S3. ML Classifiers on High Methylation Parameter – best Model describing "High Methylation"

Model	AUC	CA	F1	Precision	Recall
**Neural Network**	0.733	0.733	0.691	0.674	0.733
**Naive Bayes**	0.559	0.367	0.394	0.583	0.367
**kNN**	0.543	0.700	0.631	0.575	0.700
Random Forest	0.447	0.733	0.691	0.674	0.733
Extreme Gradient Boosting Random Forest (xgboost)	0.429	0.600	0.616	0.636	0.600
AdaBoost	0.419	0.567	0.576	0.587	0.567
Gradient Boosting (scikit-learn)	0.366	0.633	0.623	0.614	0.633
SVM	0.255	0.767	0.665	0.588	0.767

Table S4. Top 5 Model Feature ranked based on Models predicting "High Methylation" classification feature (label)

Features	Gain ratio	Gini	ANOVA	ReliefF
tLDH	0.018	0.018	1.861	0.070
wLDH	0.027	0.028	2.714	0.069
pLDH	0.006	0.005	0.956	0.050
ALH	0.018	0.017	2.325	0.048
tGGT	0.068	0.048	0.028	0.045

Table S5. ML Classifiers on "Suggested Good Quality" Parameter – best Model describing this classifier feature (label)

Model	AUC	CA	F1	Precision	Recall
Gradient Boosting (scikit-learn)	1.00	1.00	1.00	1.00	1.00
Neural Network	1.00	0.97	0.97	0.98	0.97
AdaBoost	0.75	0.97	0.96	0.97	0.97
Random Forest	1.00	0.93	0.90	0.87	0.93
kNN	0.55	0.93	0.90	0.87	0.93
Extreme Gradient Boosting Random Forest (xgboost)	0.50	0.93	0.90	0.87	0.93
SVM		0.93	0.90	0.87	0.93
Naive Bayes	0.77	0.07	0.01	0.00	0.07

Table S6. Top 5 Model Feature ranked based on Models predicting "Suggested Good Quality" classification feature (label)

Features	Gain ratio	Gini	ANOVA	ReliefF
ALH	0.070	0.024	36.977	0.050
VCL	0.069	0.024	36.019	0.047
tLDH	0.035	0.009	3.449	-0.042
VAP	0.069	0.024	8.772	-0.045
wLDH	0.076	0.029	4.930	-0.052

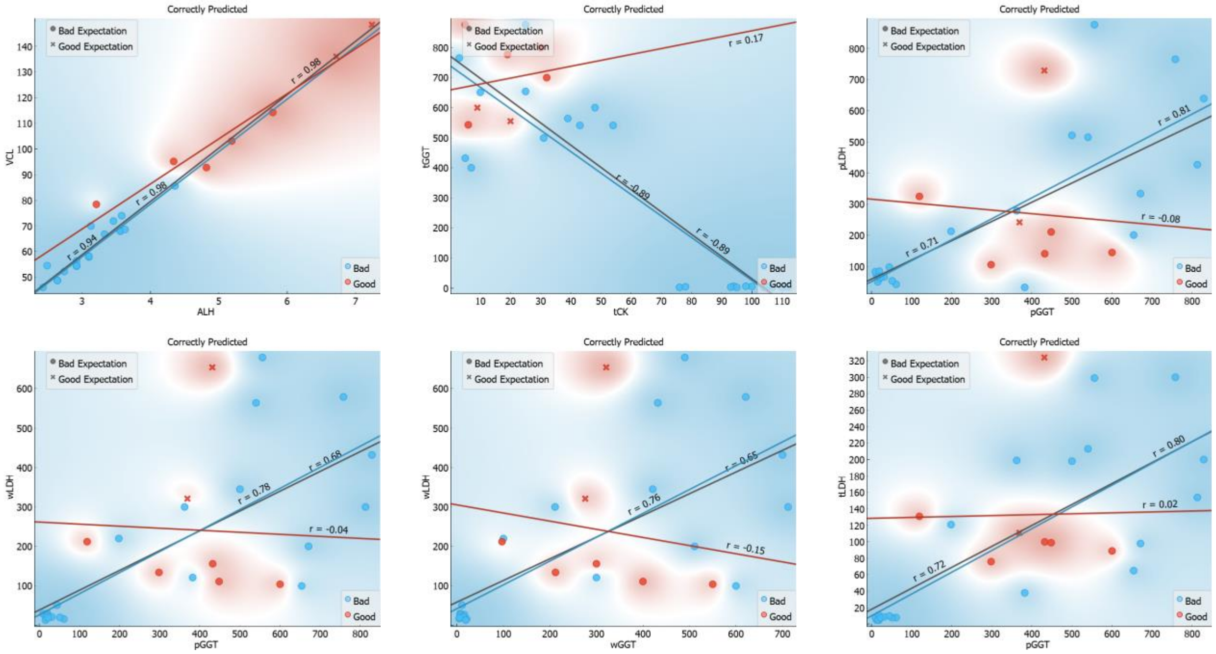

Supplementary Figure S7. *Best feature correlations in the dataset after only correctly predicted instances were selected. Data points are coloured based on classification parameter "Quality", while their shape resembles classification parameter "Suggested Good Quality".*

Overfitting testing

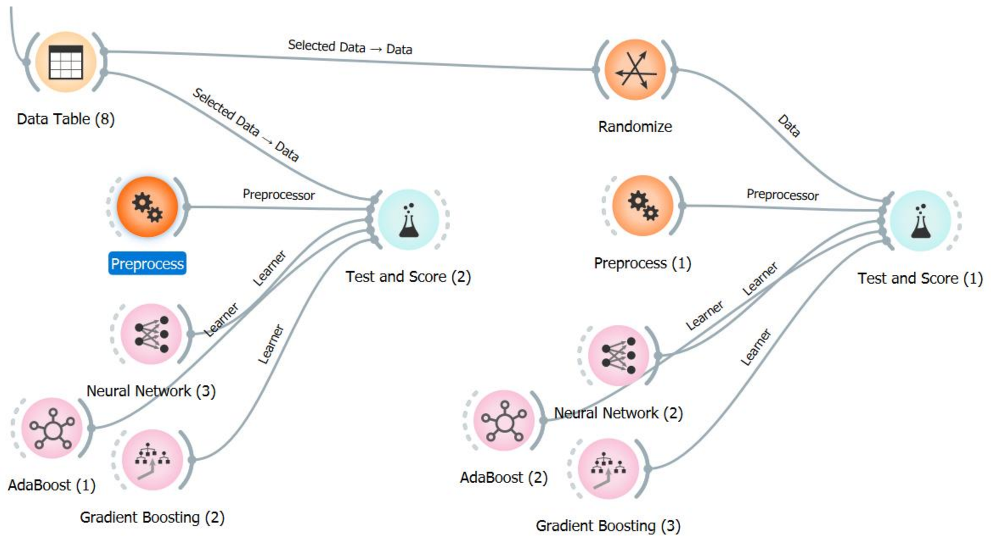

Model	AUC	CA	F1	Precision	Recall
Neural Network	0.988	0.967	0.966	0.968	0.967
Gradient Boosting (2)	0.851	0.900	0.897	0.897	0.900
AdaBoost (1)	0.835	0.900	0.897	0.897	0.900

Model	AUC	CA	F1	Precision	Recall
Neural Network	0.708	0.700	0.692	0.685	0.700
Gradient Boosting (3)	0.630	0.667	0.667	0.667	0.667
AdaBoost (2)	0.512	0.633	0.641	0.651	0.633

Supplementary Figure S8. *Overfitting testing using the methodology described by Demšar and Zupan (2021) in Plos ONE Computational Biology (Demšar J, Zupan B (2021) Hands-on training about overfitting. PLOS Computational Biology 17(3): e1008671. https://doi.org/10.1371/journal.pcbi.1008671). The overfitting test is implemented directly in Orange as in the paper. The SLP-NN, AdaBoost and Gradient Boosting were linked to Test and Score widget that performs cross-validation in 2-5-10 folds. Preprocess widget selects first 10 features from the dataset based on information gain. Randomize widget was set to scramble the classification features only before feeding the dataset to the Test and Score widget and SLP-NN, AdaBoost and Gradient Boosting models. "In this workflow pre-processing is not done prior to splitting the data. The pre-processing recipe, provided by the Preprocess widget, enters the cross-validation procedure, and is applied to each training data subset separately, without being informed by the data that is used in testing" (Demšar J et al., 2021).*

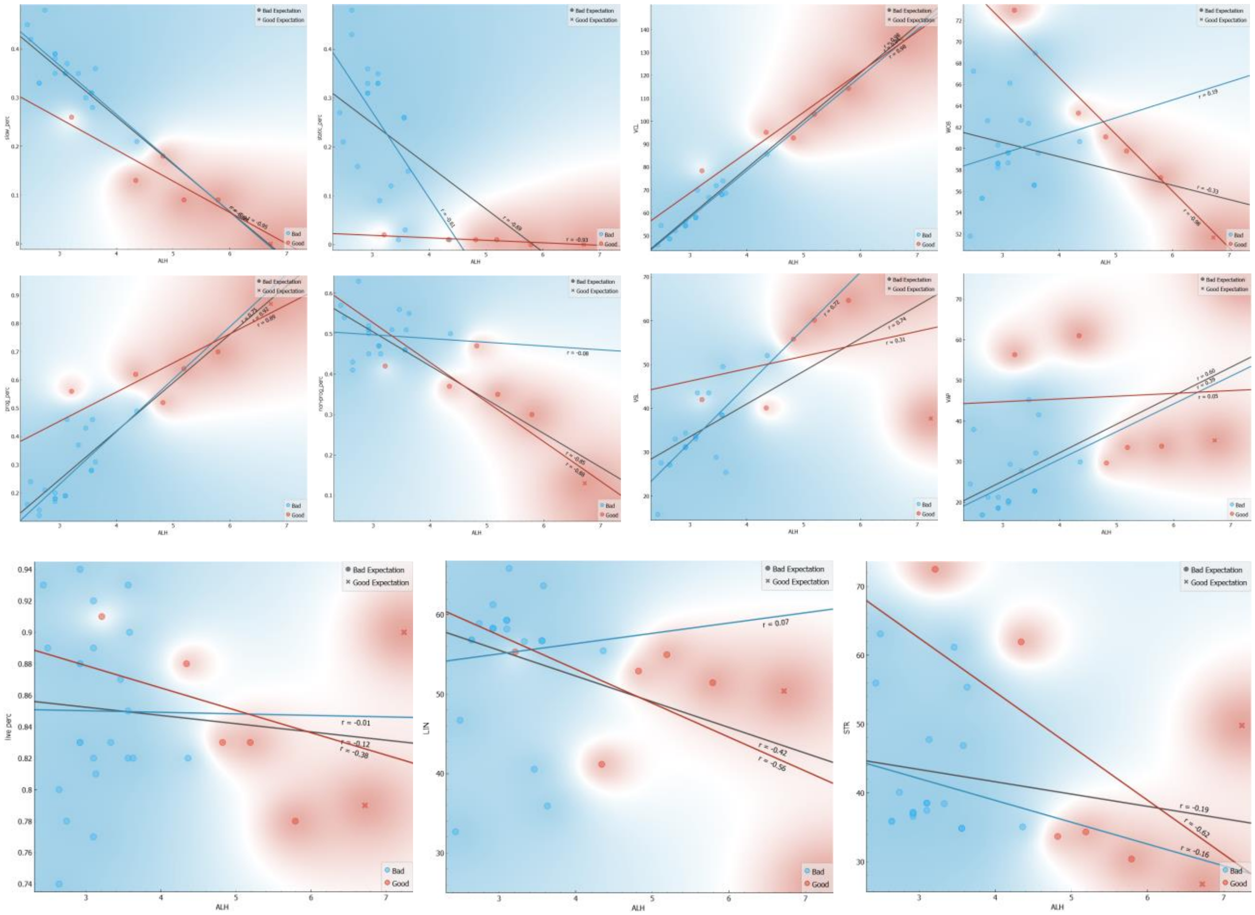

Supplementary Figure S9. *Correlation between ALH data and other CASA parameters, as well as between the ALH and vitality parameters, after only correctly predicted for “Suggested Good Quality” classification feature samples were selected.*
