# Supplementary figures and images for: Machine Learning Approach for Muscovy Duck (Cairina moschata) Semen Quality Assessment
Source: Animals (Basel). 2023 May 10;13(10):1596. doi: 10.3390/ani13101596 (PMC10215291; doi:10.3390/ani13101596)

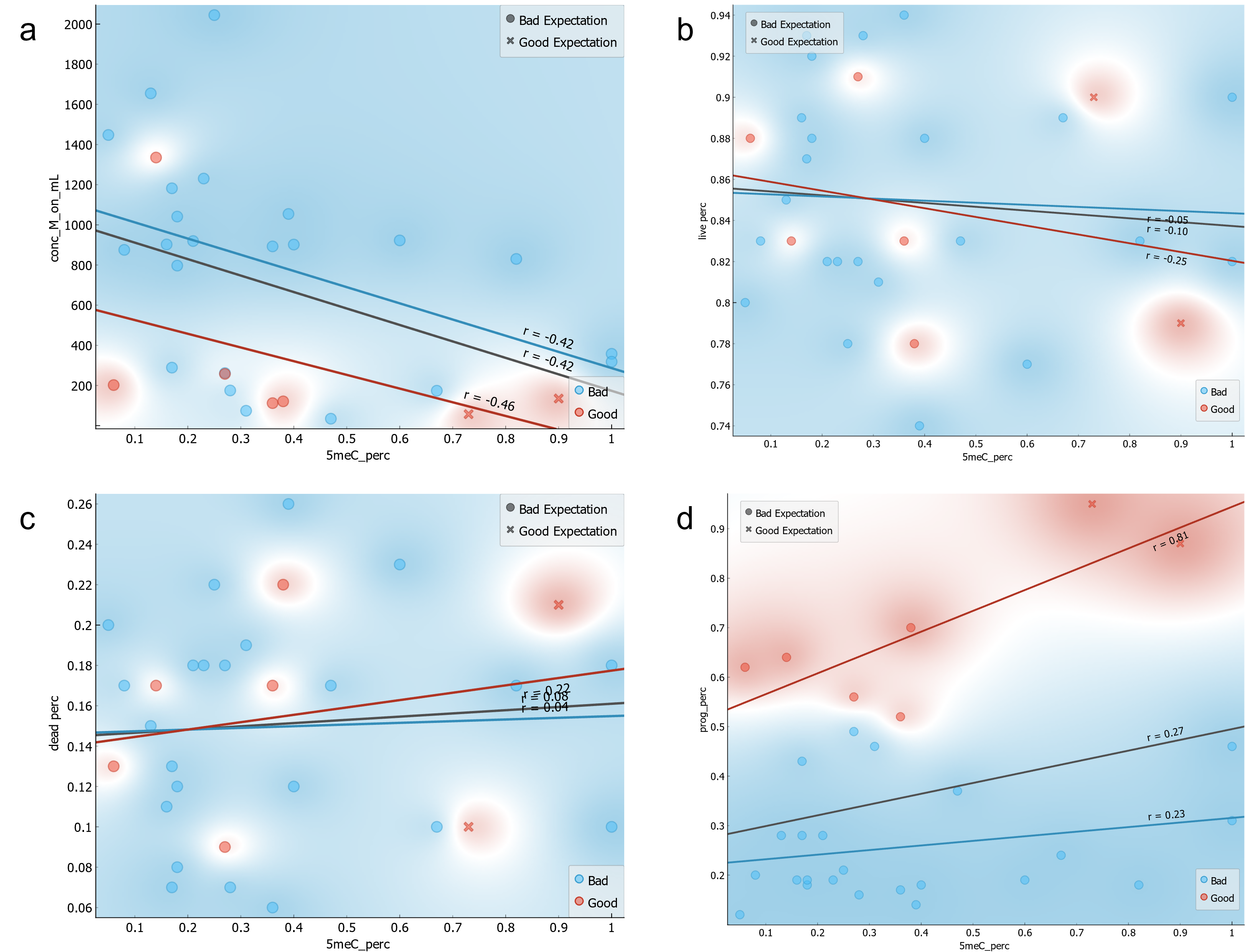

Supplement: Supplementary file 1 [file animals-13-01596-s001.zip › Supplementary Figure S1.tif]

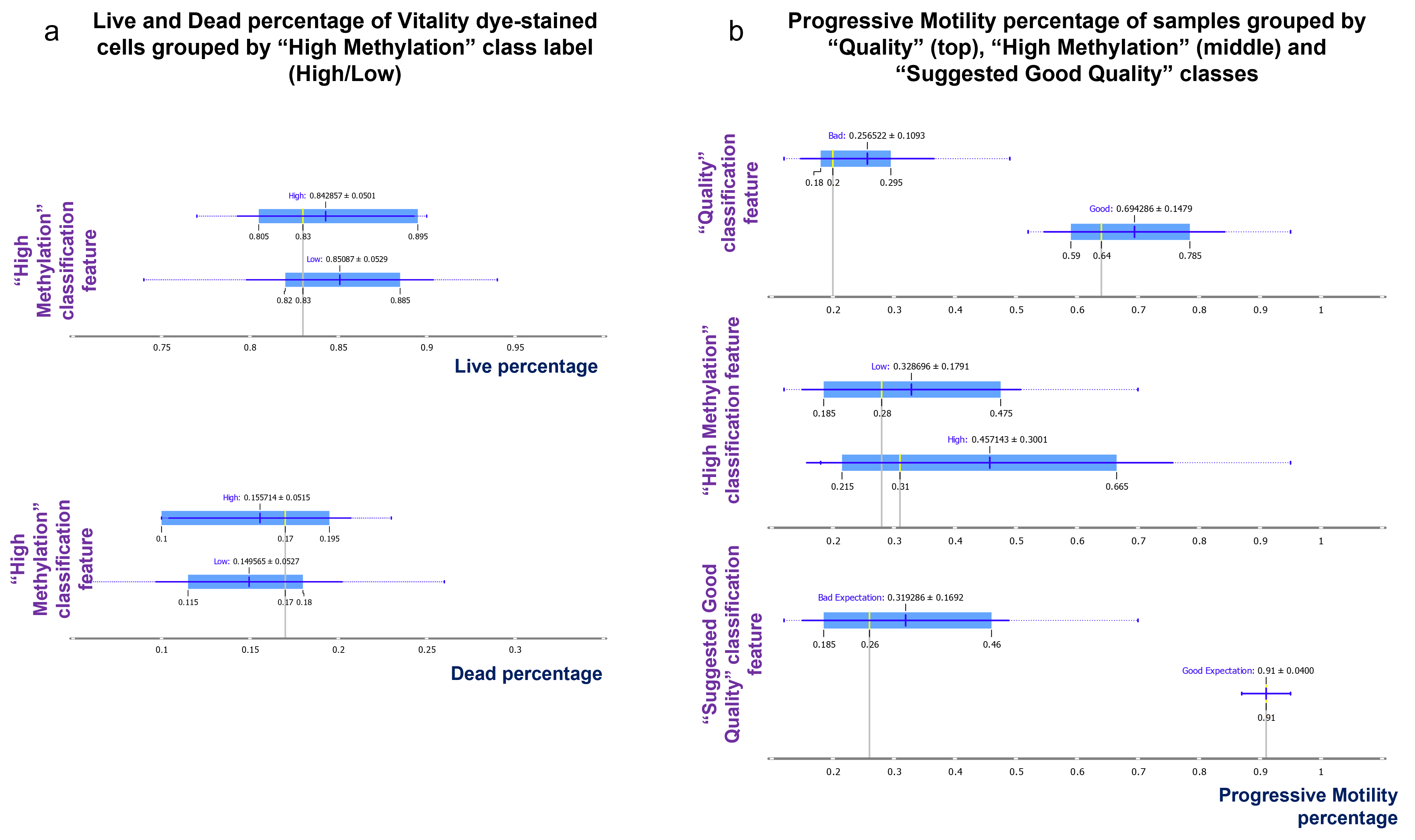

Supplement: Supplementary file 1 [file animals-13-01596-s001.zip › Supplementary Figure S2.tif]

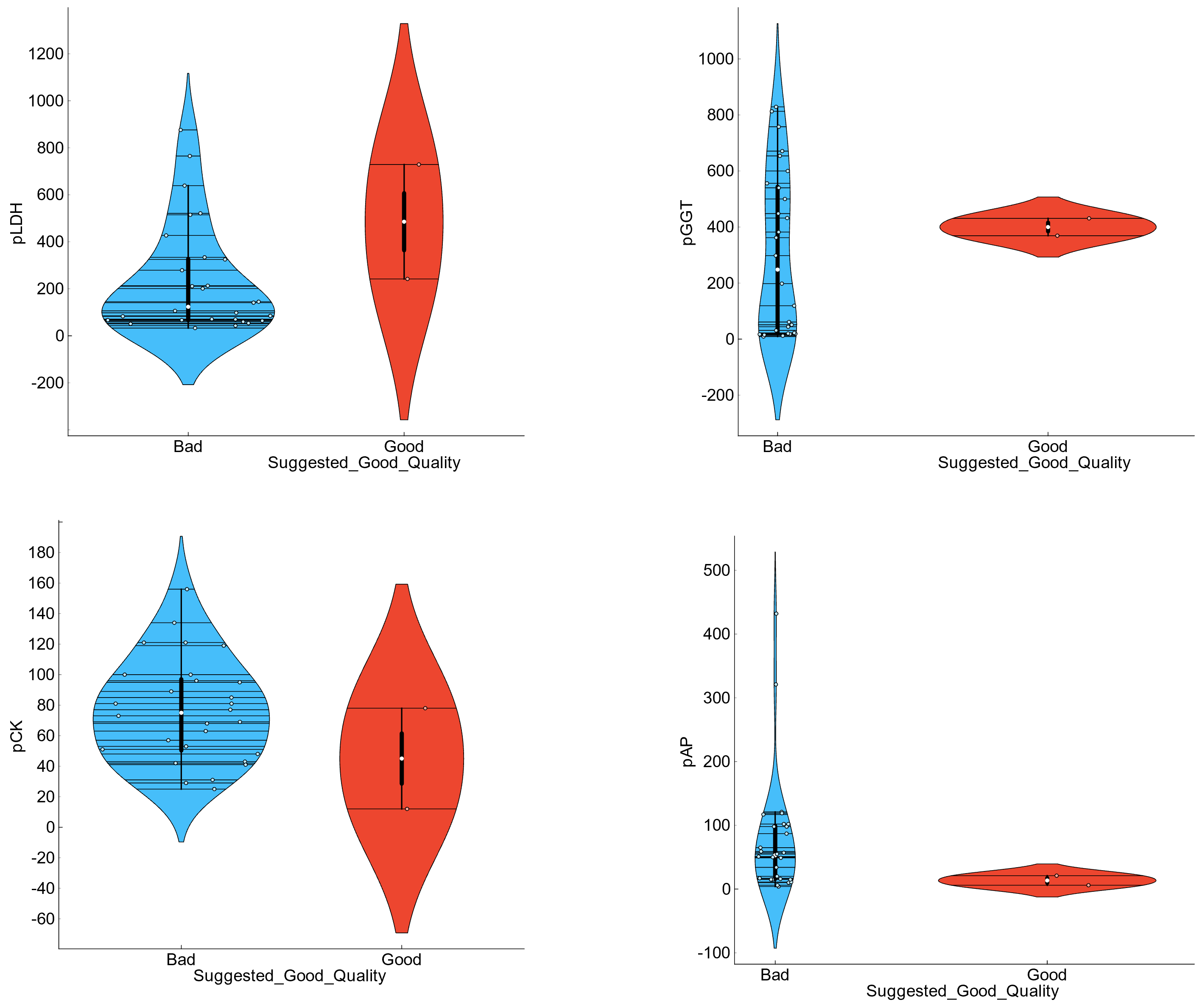

Supplement: Supplementary file 1 [file animals-13-01596-s001.zip › Supplementary Figure S3.tif]

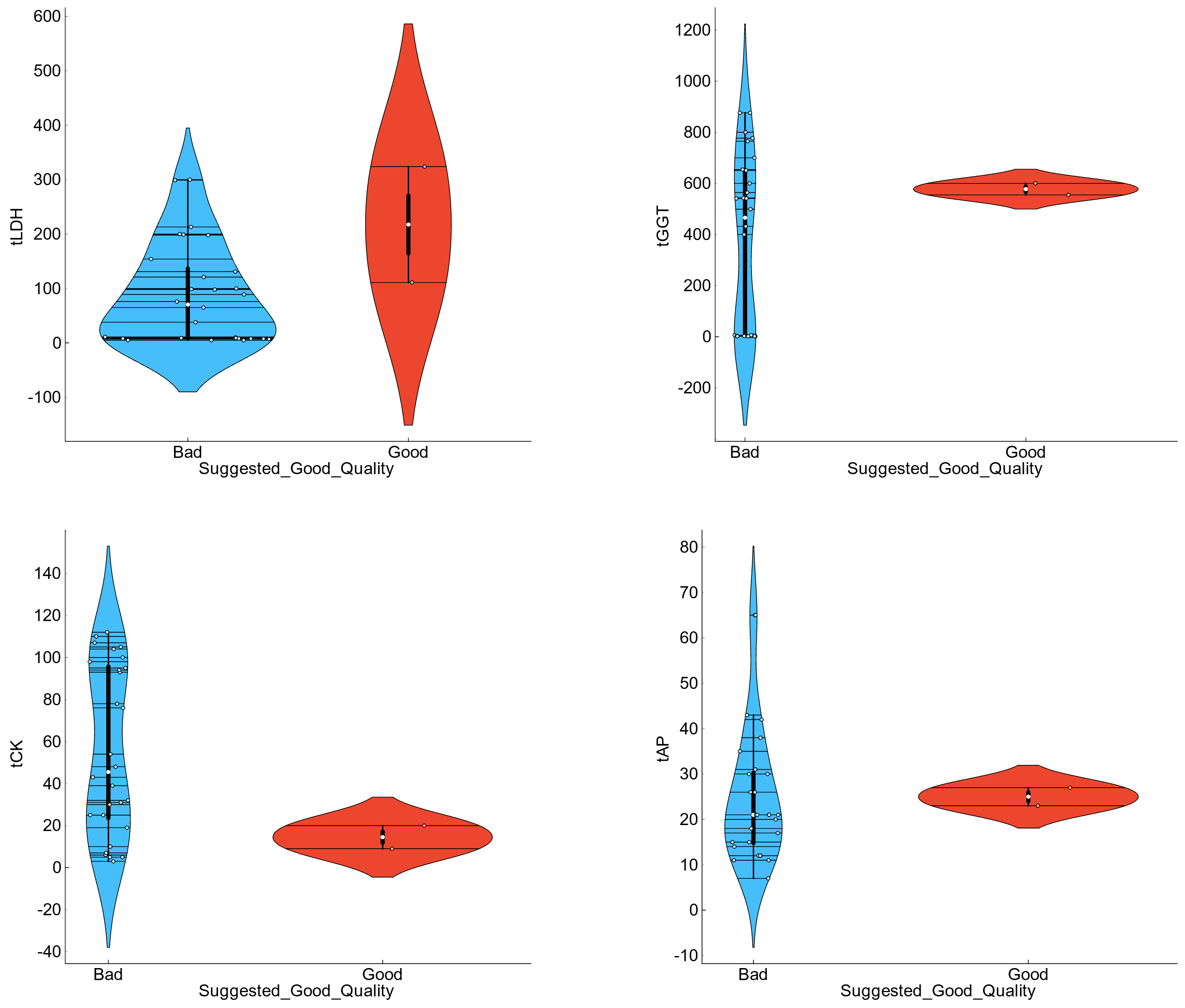

Supplement: Supplementary file 1 [file animals-13-01596-s001.zip › Supplementary Figure S4.tif]

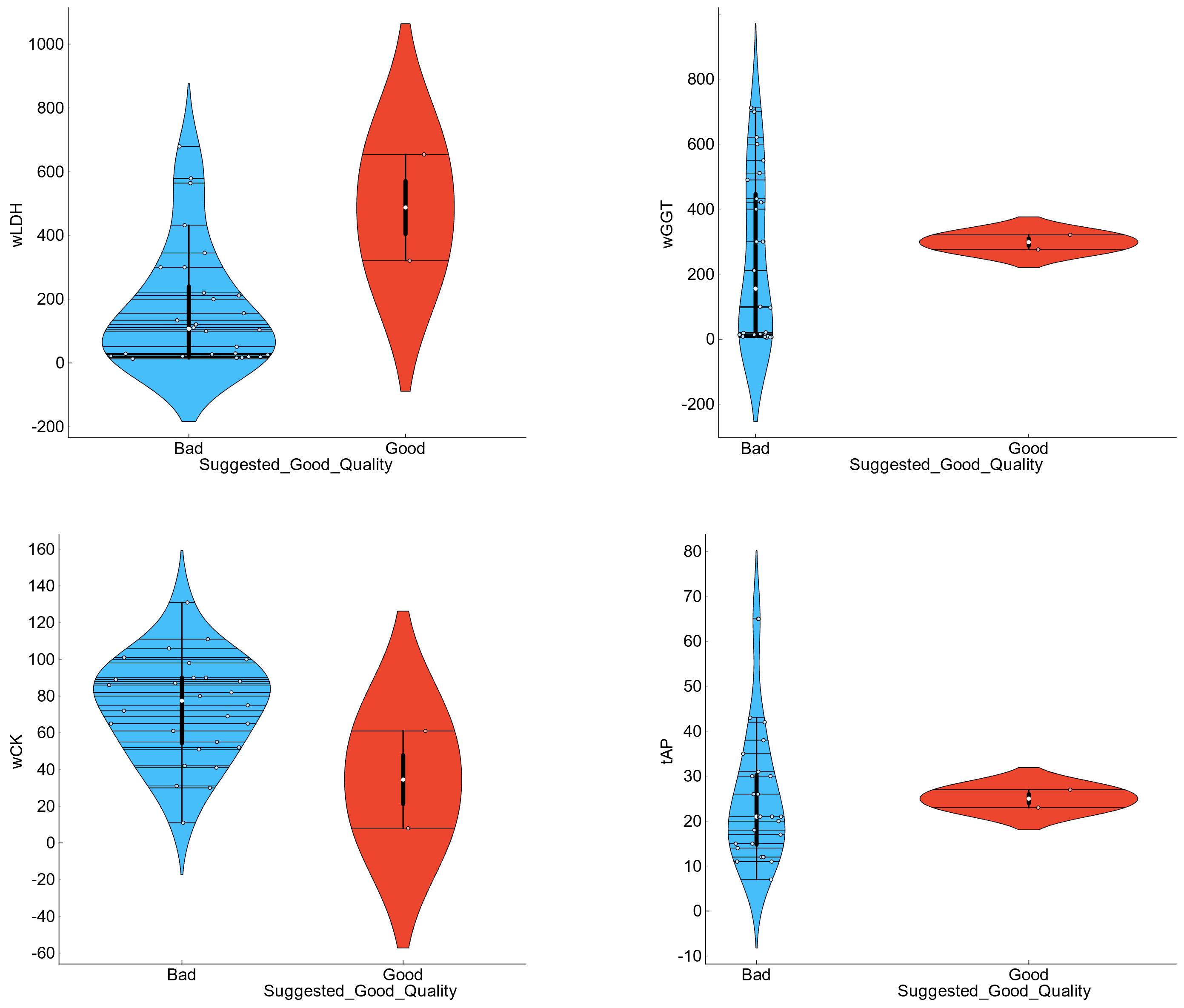

Supplement: Supplementary file 1 [file animals-13-01596-s001.zip › Supplementary Figure S5.tif]

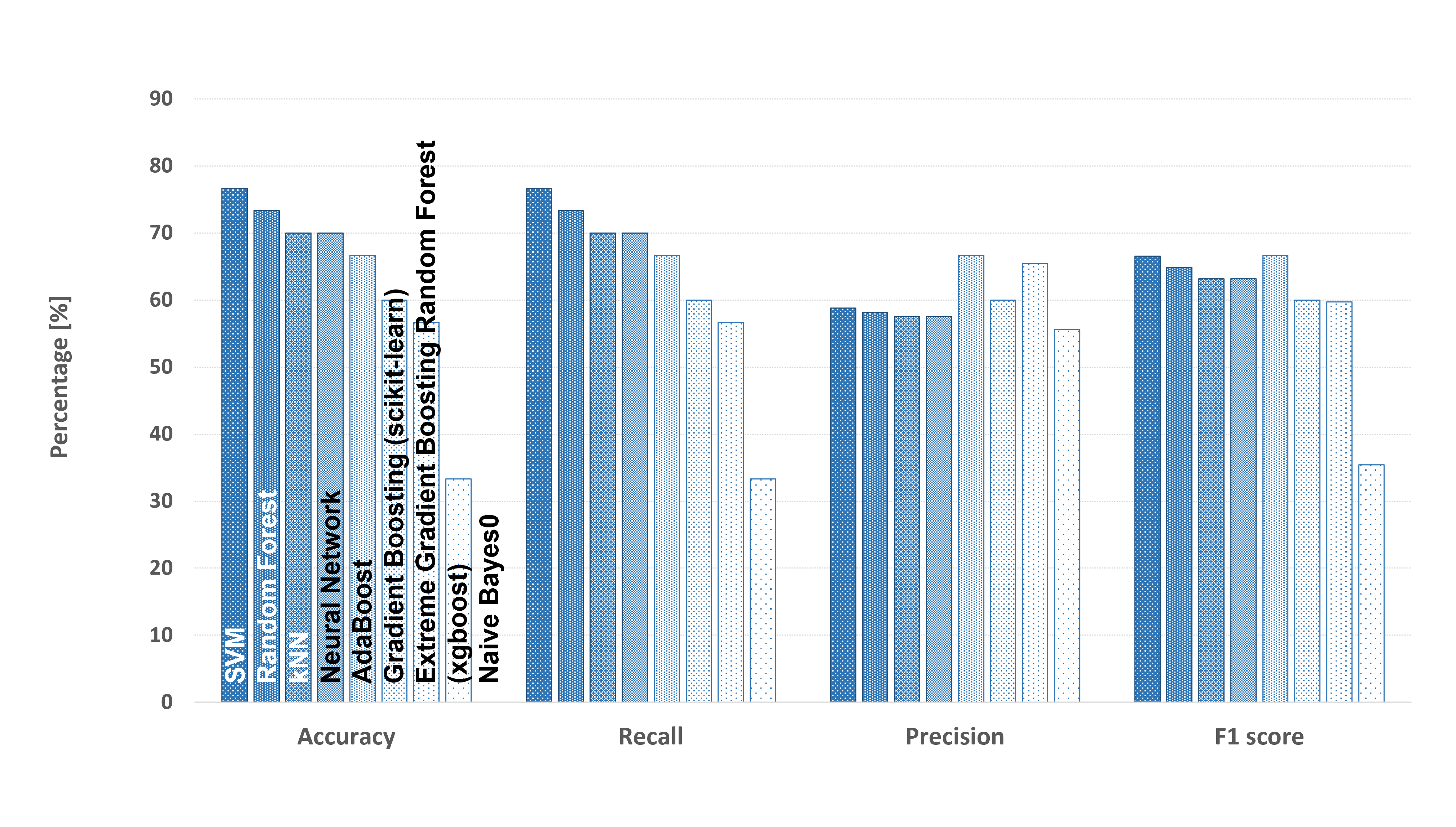

Supplement: Supplementary file 1 [file animals-13-01596-s001.zip › Supplementary Figure S6.tif]

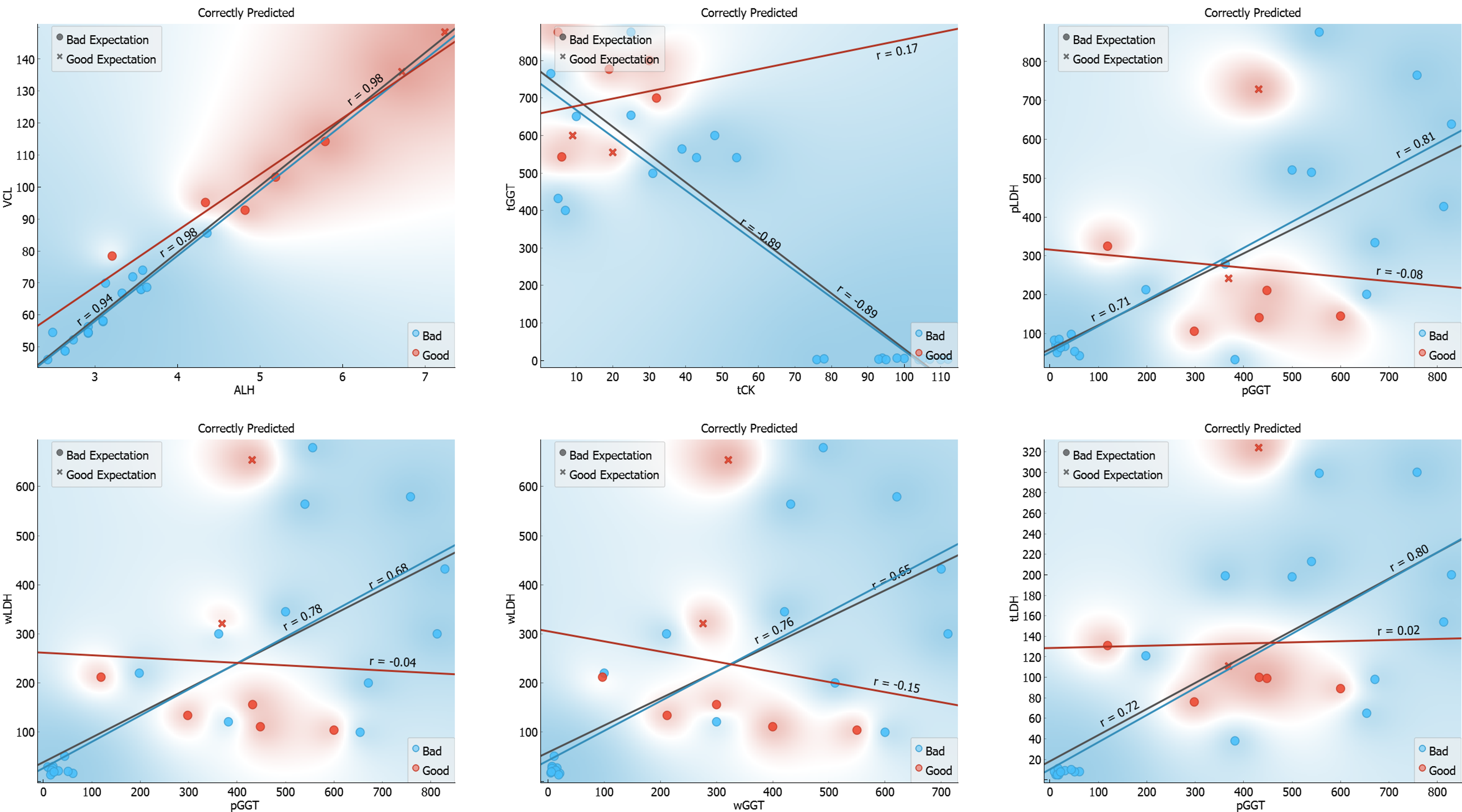

Supplement: Supplementary file 1 [file animals-13-01596-s001.zip › Supplementary Figure S7.tif]

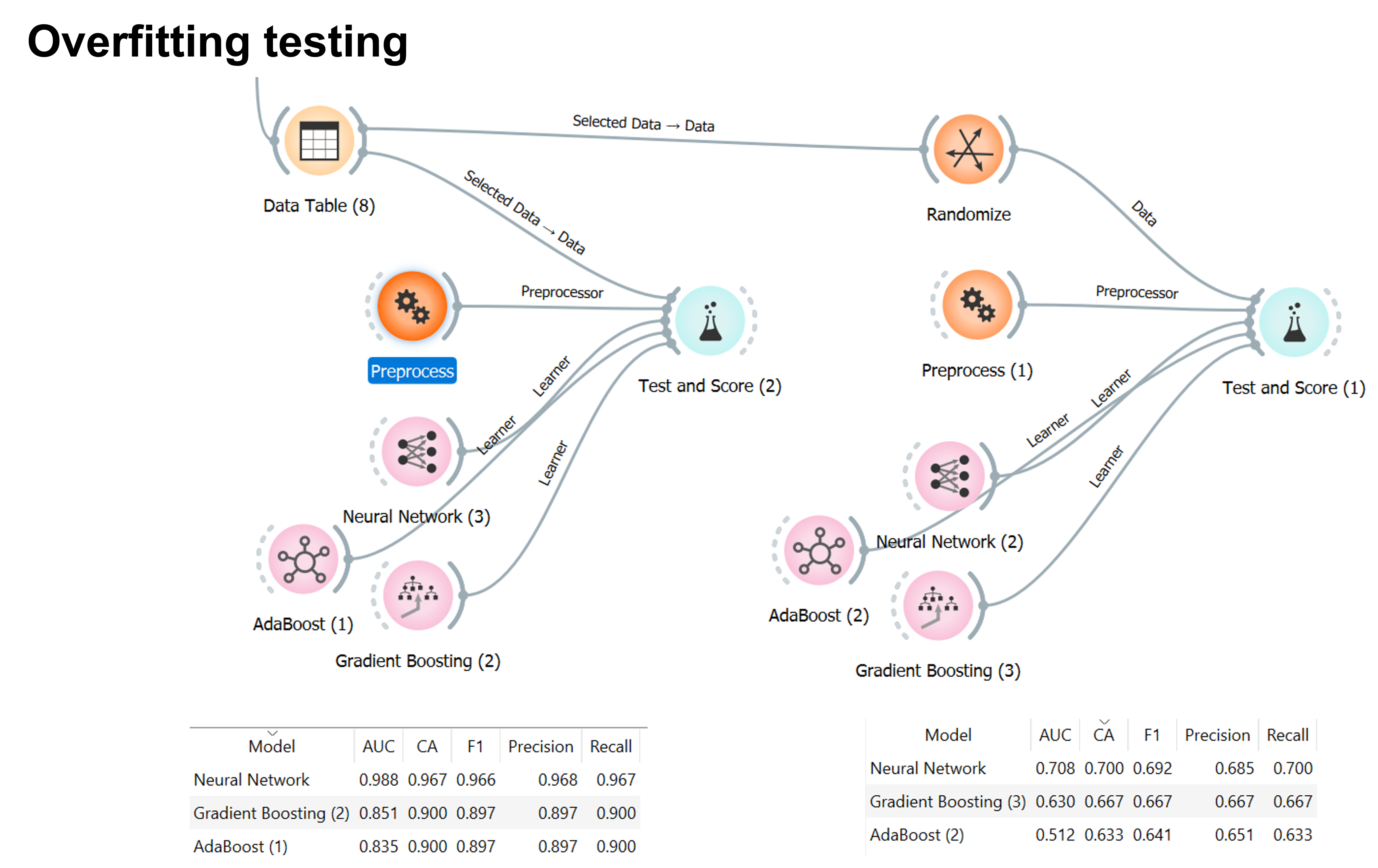

Supplement: Supplementary file 1 [file animals-13-01596-s001.zip › Supplementary Figure S8.tif]

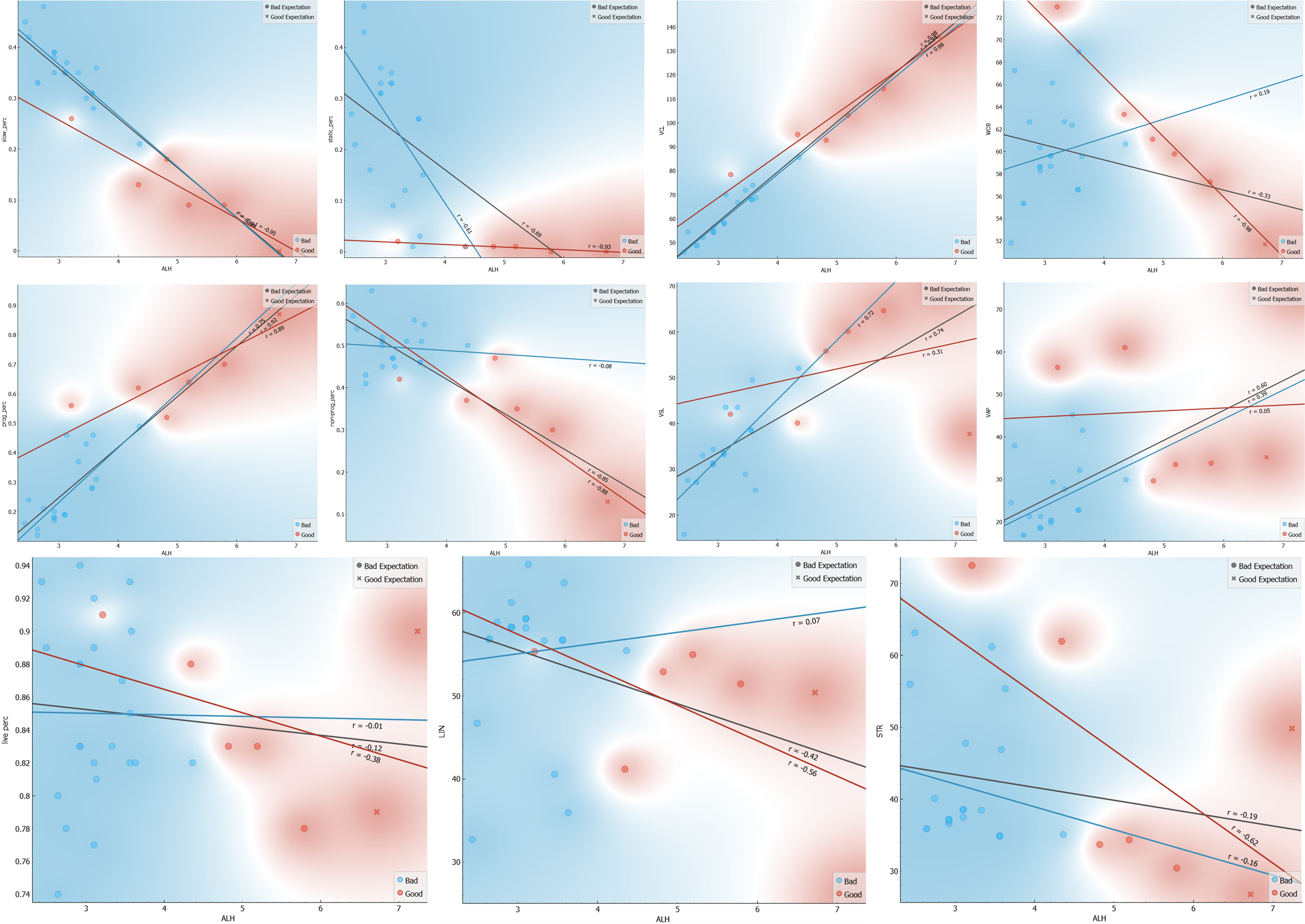

Supplement: Supplementary file 1 [file animals-13-01596-s001.zip › Supplementary Figure S9.tif]

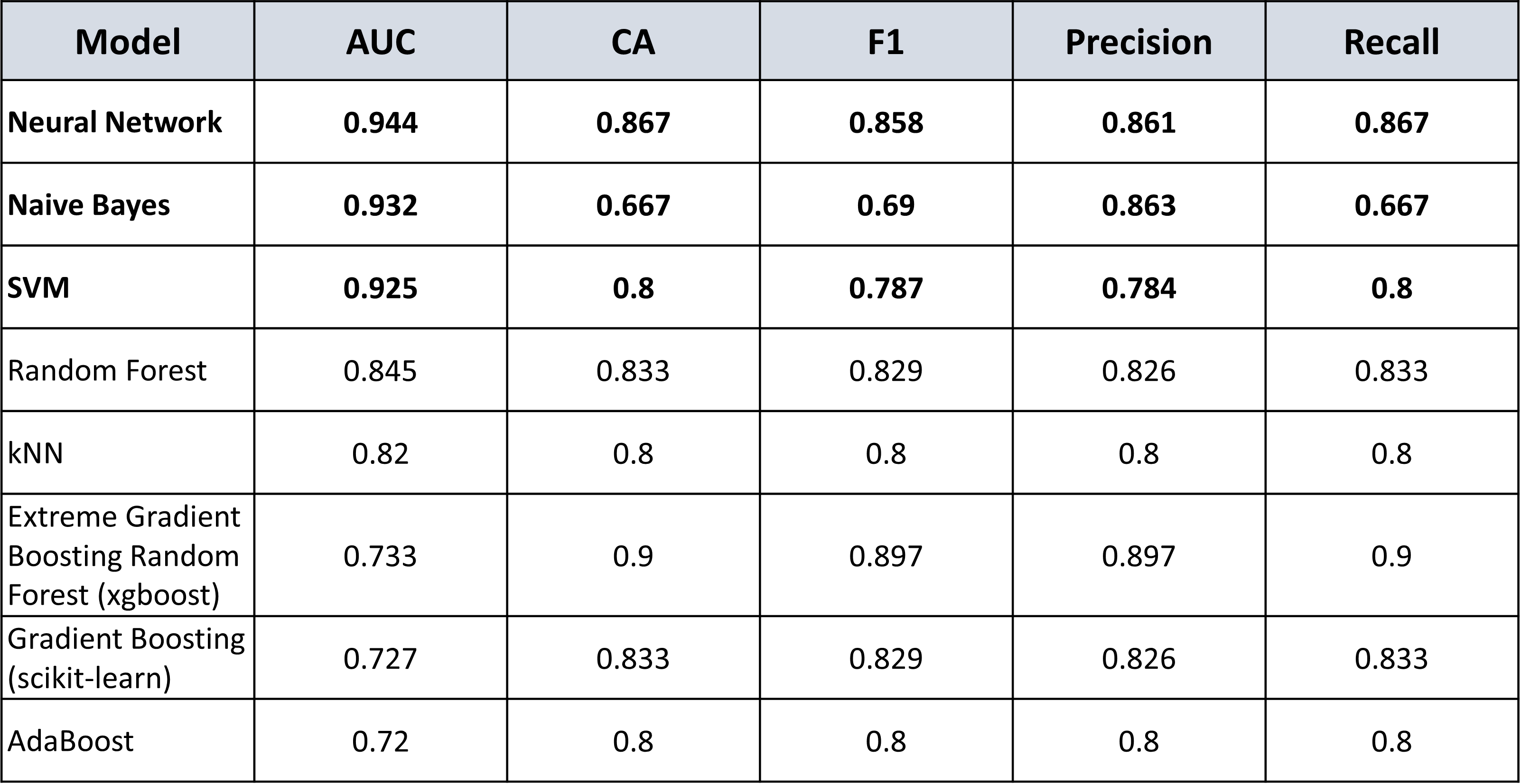

Supplement: Supplementary file 1 [file animals-13-01596-s001.zip › Table S1.tif]

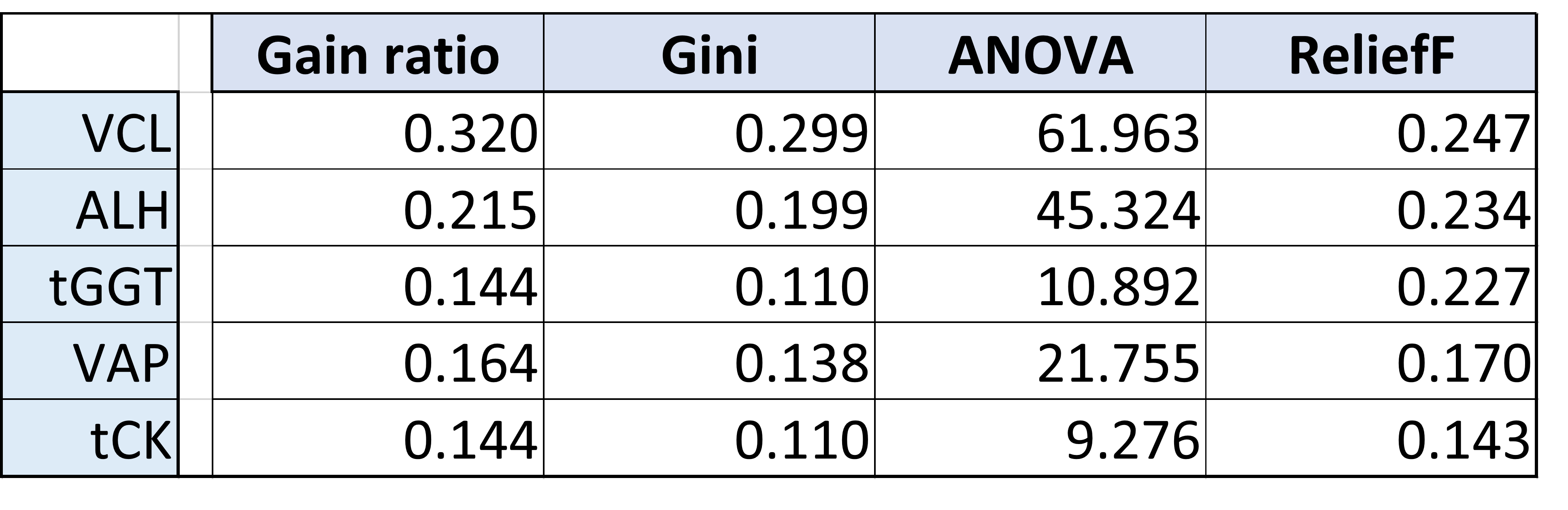

Supplement: Supplementary file 1 [file animals-13-01596-s001.zip › Table S2.tif]

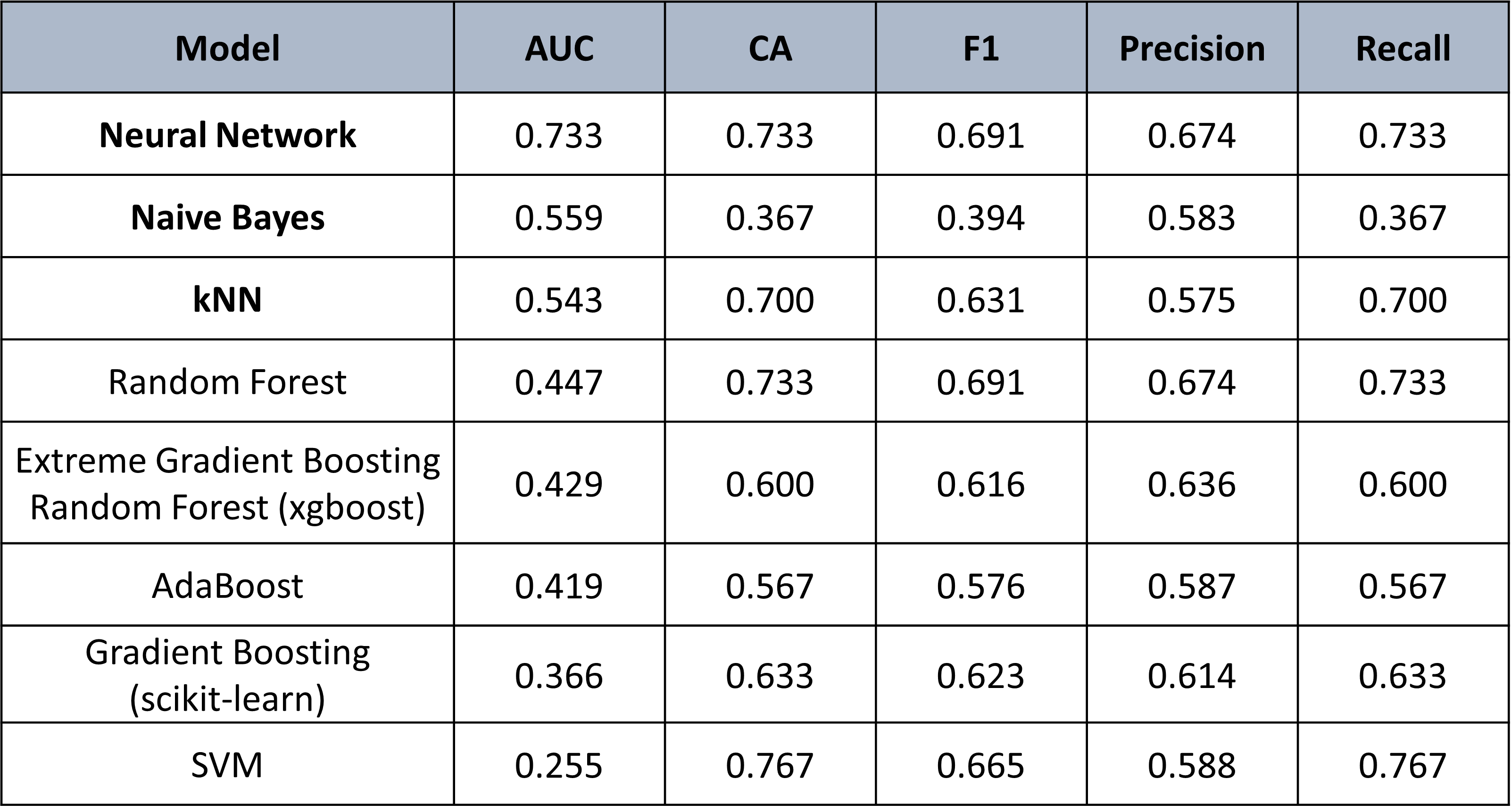

Supplement: Supplementary file 1 [file animals-13-01596-s001.zip › Table S3.tif]

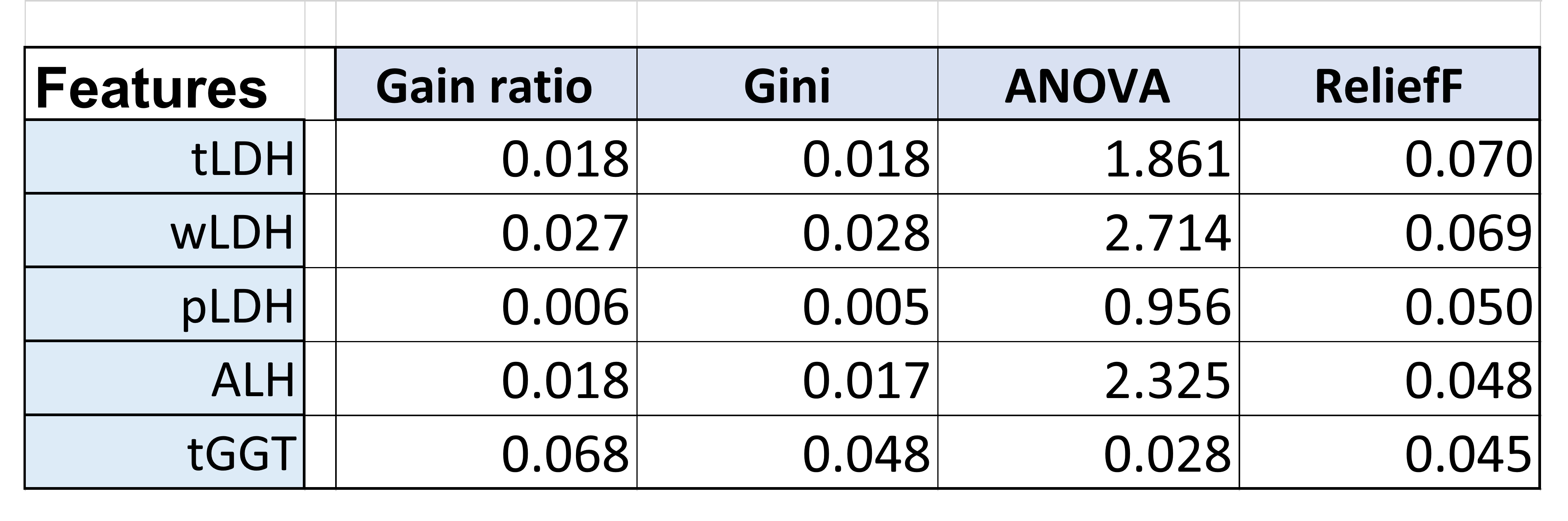

Supplement: Supplementary file 1 [file animals-13-01596-s001.zip › Table S4.tif]

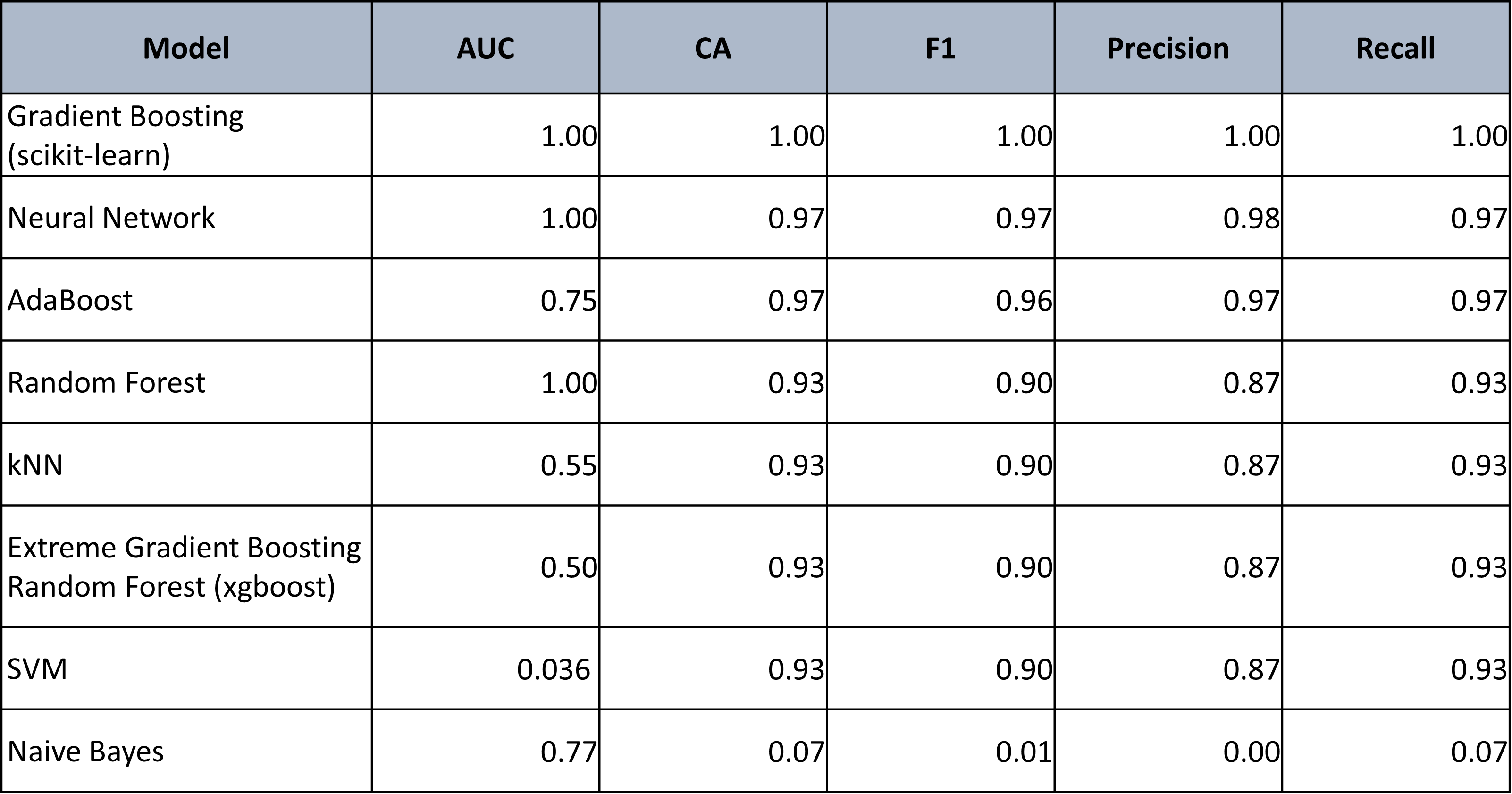

Supplement: Supplementary file 1 [file animals-13-01596-s001.zip › Table S5.tif]

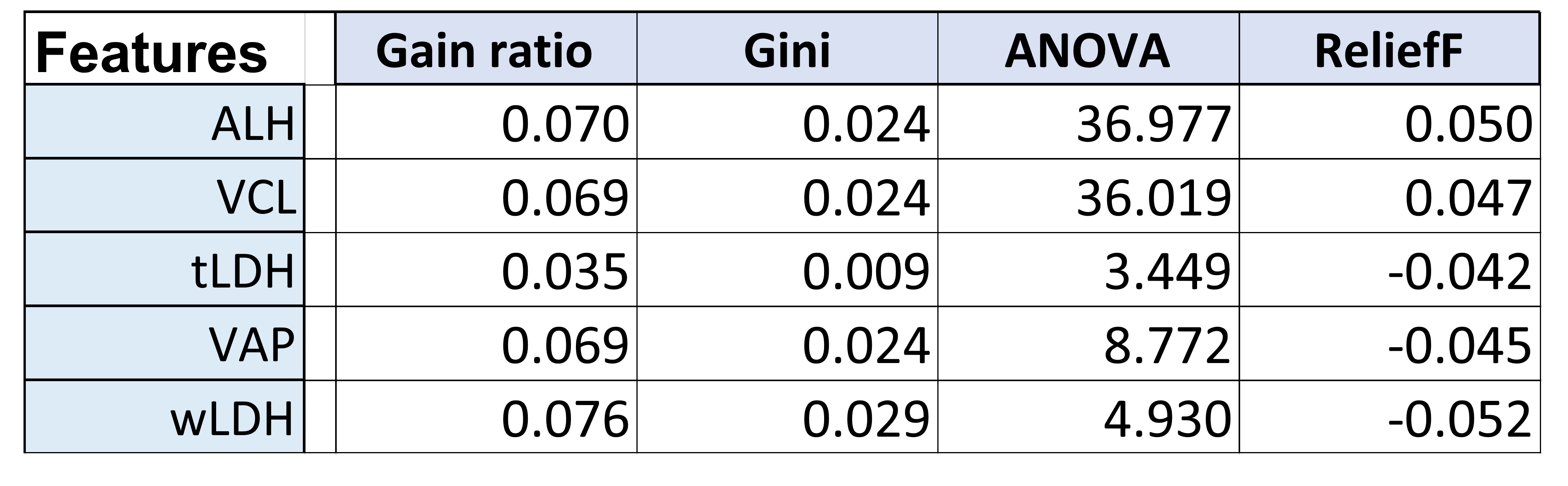

Supplement: Supplementary file 1 [file animals-13-01596-s001.zip › Table S6.tif]
